# Concentrations of glutamate and *N*-acetylaspartate detected by magnetic resonance spectroscopy in the rat hippocampus correlate with hippocampal-dependent spatial memory performance

**DOI:** 10.3389/fnmol.2024.1458070

**Published:** 2024-08-16

**Authors:** João M. N. Duarte

**Affiliations:** ^1^Diabetes and Brain Function Unit, Department of Experimental Medical Science, Faculty of Medicine, Lund University, Lund, Sweden; ^2^Wallenberg Centre for Molecular Medicine, Lund University, Lund, Sweden

**Keywords:** neurochemicals, metabolites, neurotransmitters, glutamate, GABA, NAA

## Abstract

Magnetic resonance spectroscopy (MRS) has been employed to investigate brain metabolite concentrations *in vivo*, and they vary during neuronal activation, across brain activity states, or upon disease with neurological impact. Whether resting brain metabolites correlate with functioning in behavioral tasks remains to be demonstrated in any of the widely used rodent models. This study tested the hypothesis that, in the absence of neurological disease or injury, the performance in a hippocampal-dependent memory task is correlated with the hippocampal levels of metabolites that are mainly synthesized in neurons, namely *N*-acetylaspartate (NAA), glutamate and GABA. Experimentally naïve rats were tested for hippocampal-dependent spatial memory performance by measuring spontaneous alternation in the Y-maze, followed by anatomical magnetic resonance imaging (MRI) and magnetic resonance spectroscopy (MRS) in the hippocampus and cortex. Memory performance correlated with hippocampal concentrations of NAA (*p* = 0.024) and glutamate (*p* = 0.014) but not GABA. Concentrations of glutamate in the cortex also correlated with spatial memory (*p* = 0.035). In addition, memory performance was also correlated with the relative volume of the hippocampus (*p* = 0.041). Altogether, this exploratory study suggests that levels of the neuronal maker NAA and the main excitatory neurotransmitter glutamate are associated with physiological functional capacity.

## 1 Introduction

The regional neurochemical profile measured non-invasively by ^1^H magnetic resonance spectroscopy (MRS) is specific to the tissue’s metabolic status, and also vary with tissue development, differentiation and injury (reviewed in Duarte et al., 2012). Over the last decades, the development of powerful methods that allow increasing resolution and sensitivity of both ^1^H MRS and imaging modalities resulted in studies that provided evidence for a relation between brain function and metabolite levels, particularly of the neurotransmitters glutamate and GABA that ubiquitously modulate the balance between inhibition and excitation in the cortex. While local GABA concentrations are generally negatively related to stimulus-induced cortical activity, positive correlation between glutamate concentrations and inter-regional activity relationships has been reported (Duncan et al., 2014). Moreover, the strength of motor network-level resting functional connectivity were found to be inversely related with local GABA concentration (Stagg et al., 2014), and cortical concentrations of both glutamate and GABA were found to be associated with the strength of the default mode network (Kapogiannis et al., 2013).

Several ^1^H MRS in the human cortex during visual or motor stimulation showed a robust activation-induced increased glutamate concentration (e.g., Mangia et al., 2007; Lin et al., 2012; Schaller et al., 2014; Apšvalka et al., 2015; Ip et al., 2017; Martínez-Maestro et al., 2019; DiNuzzo et al., 2022; see also the recent review by Koush et al., 2022). This glutamate increase has been confirmed during visual or somatosensory stimulation in animal models (e.g., Sonnay et al., 2017, 2018; Seuwen et al., 2019) and consistently reproduced across many stimulation paradigms and MRS acquisition modes in rodents (Just, 2021). Cortical stimulation has been found to induce a reduction of GABA levels, although the opposite or no change has been reported in several studies (discussed in Koush et al., 2022). In awake mice, somatosensory stimulation induced an increase in the cortical concentrations of both glutamate and GABA, although the later at a slower rate (Takado et al., 2022). However, this is the only report of GABA levels increasing with direct neuronal stimulation. Increase in levels of glutamate plus glutamine (the so called Glx) but not of GABA were found to increase in the dorsolateral prefrontal cortex during working memory demand in humans (Oh et al., 2024). It has also been reported that concentrations of glutamate and GABA in the human cortex decrease during negative blood-oxygen-level-dependent (BOLD) responses in functional magnetic resonance imaging (MRI), that is, during neuronal deactivation (Boillat et al., 2020). Reduced levels of *N*-acetylaspartate (NAA) and increased levels *N*-acetylaspartatylglutamate (NAAG) were also reported to occur during visual stimulation (Landim et al., 2016), in line with a role of NAAG as neurotransmitter. Reduced NAA signal was also found to decrease in the rat prefrontal cortex during chemogenic stimulation (Baslow et al., 2016).

A plethora of MRS studies on patients with neurological disorders reported that certain neurochemicals correlate with measures of disease severity, namely the neuronal marker NAA (Duarte et al., 2013). Moreover, several neurochemical profile modifications are observable in the brain by MRS during healthy aging (e.g., Duarte et al., 2014; Fowler et al., 2021). However, direct tests of the predictive capabilities of ^1^H MRS are scarce in the literature. Of particular interest is a study suggesting that the level of some neurometabolites in the occipital cortex, namely glutamate and choline (relative to levels of creatine), measured in children during the critical period in which the neurocircuits that support skilled reading are developing can predict reading performance a couple of years later (Pugh et al., 2014). However, a handicap of this study is that metabolite levels were reported as ratio to creatine signals (internal reference). At the age of these subjects and until adulthood, levels of brain metabolites including creatine suffer substantial variations (Blüml et al., 2013). Brain creatine variations during development have been also reported in animal models using high-resolution MRS (e.g., Kulak et al., 2010; Morgan et al., 2013). Moreover, Pugh et al. did not separate the signals of glutamine from those of glutamate, and a combination of both was detected, the so called “Glx” (Pugh et al., 2014). In a recent study, NAA levels in the dorsal anterior cingulate cortex were found to correlate with emotional and affective traits in healthy young adults (White et al., 2021). In this study, moderate correlations were also observed between emotional traits and choline, and between Glx and behavior flexibility.

Apart from NAA (Patel et al., 2014), relations between concentrations of neurochemicals and behavior or cognition have hardly found any convincing support in non-pathological subjects. Thus, taking advantage of the high magnetic field MRS in small rodent experiments, we now tested the hypothesis that components of the neurochemical profile that are primarily synthetized in neurons—NAA, glutamate and GABA—are correlated with memory performance in healthy rats.

## 2 Methods

All experiments were approved by the local ethics committee (Service de la Consommation et des Affaires Vétérinaires, Epalinges, Switzerland; #VD2610). Experimentally naive male Wistar rats at 2 months of age (307 ± 33 g, *n* = 30, out-bred strain from Charles River Laboratoires, France) were housed on a 12-h light–dark cycle with room temperature at 22°C and humidity at 60%. Food (Kliba Nafag 3800 from Provimi Kliba, Kaiseraugst, Switzerland) and water were provided *ad libitum*. Behavior analyses were performed at least 1 week after arrival from the supplier, and MR scans were performed the following day. The delay between behavior testing and MR scans was 16–24 h.

### 2.1 Y-maze task

The behavioral analyses were adapted from those previously reported for mice (Duarte et al., 2012). Spontaneous alternation was measured in a Y-maze with three arms measuring 35 cm long, 9 cm wide and 30 cm height, and converging to equal angles. The animals were placed at the bottom of one arm in the Y-maze and allowed to explore freely all three arms for a single 8-min session in the dark. The measured spontaneous alternation behavior was used to assess hippocampal-dependent spatial memory (Lalonde, 2002). Complete spontaneous alternation was defined as successive entries into the three arms and expressed as fraction of the possible alternations in the respective test. The number of entries in the arms of the maze allowed accessing locomotor activity and exploratory behavior.

### 2.2 MRS and MRI

MRS and MRI were carried out in a horizontal 14.1 T/26 cm magnet (Magnex Scientific, Abingdon, UK), with a 12 cm inner-diameter gradient (400 mT/m in 200 ms, minimized eddy currents), interfaced with a DirectDrive console (Agilent Technologies, Palo Alto, CA, United States). Radio frequency transmission and reception was achieved with a home-built quadrature surface coil composed of two geometrically decoupled single-turn loops of 12 mm inner diameter resonating at 600 MHz.

**Figure 1:**
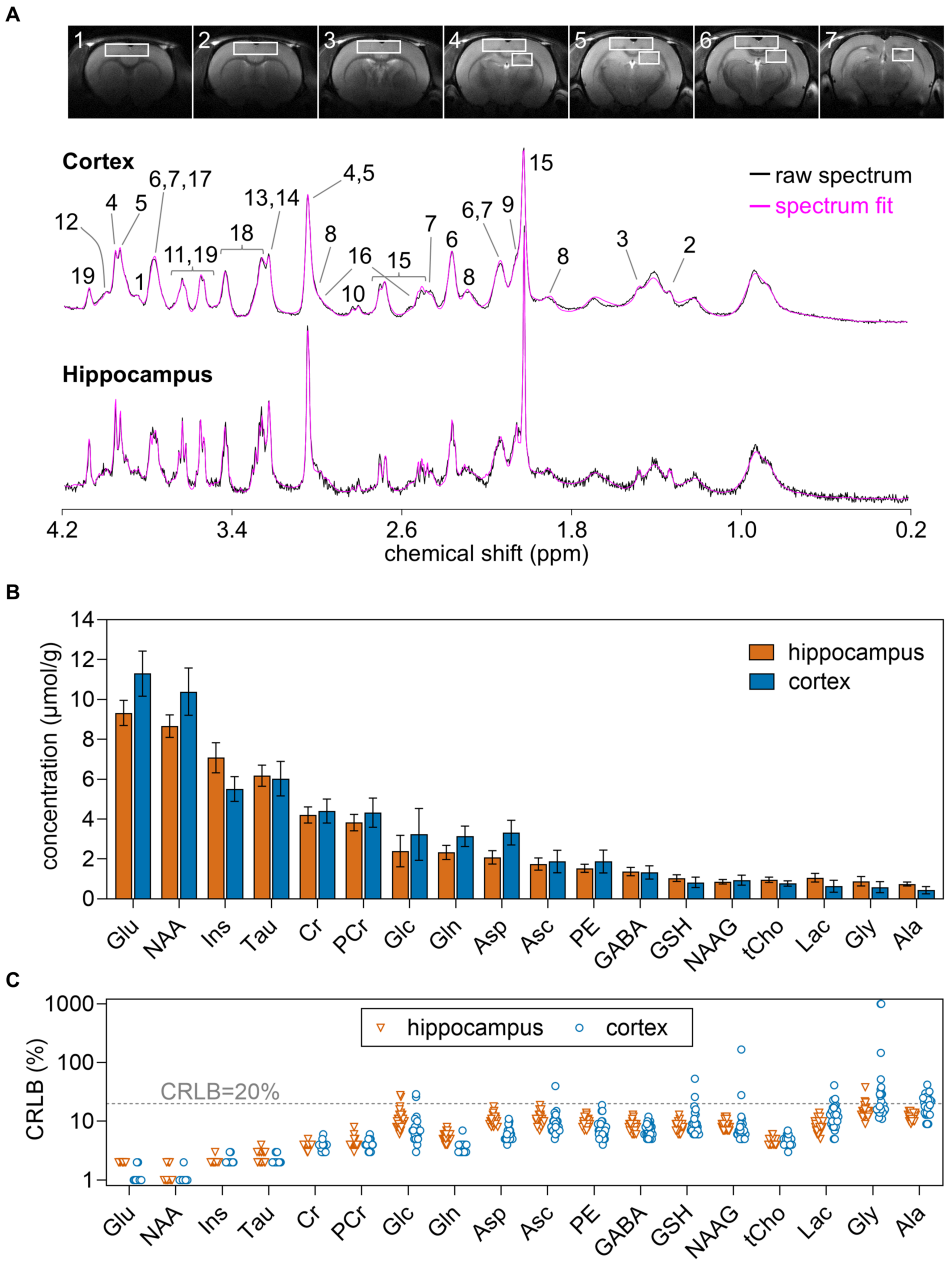
**(A)** Typical VOIs used for MRS overlaid on 8-mm MRI slices of the rat brain, and representative ^1^H spectra acquired *in vivo* at 14.1 T from the rat dorsal hippocampus and cortex. The pink line overlaid over each spectrum is the respective LCmodel fit result. **(B)** Concentration of metabolites measured in the hippocampus and cortex using MRS at 14.1 T. **(C)** CRLB values resulting from LCModel fitting. Concentrations are shown as mean ± SD of *n* = 30, while individual measurements are shown for CRLB. Peak assignment in **(A)** is as follows: 1, glucose; 2, lactate; 3, alanine; 4, phosphocreatine; 5, creatine; 6, glutamate; 7, glutamine; 8, GABA; 9, NAAG; 10, aspartate; 11, glycine; 12, phosphorylethanolamine; 13, phosphrylcholine; 14, glycerylphosphorylcholine; 15, NAA; 16, glutathione; 17, ascorbate; 18, taurine; 19, *myo*-inositol.

Rats were anesthetized with 1–2% isoflurane in O_2_ and stereotaxically fixed. Body temperature was maintained at 37°C by warm water circulation. Anesthesia level was adjusted to maintain a ventilation rate around 70 breaths per minute. All MR protocols were performed as detailed previously (Duarte et al., 2009). Briefly, field homogeneity in the regions of interest was achieved with FAST(EST)MAP (Gruetter, 1993; Gruetter and Tkác, 2000), T_2_-weighted MRI was performed with a fast-spin-echo sequence with repetition time of 4 s and echo time of 40 ms, and MRS in the hippocampus and cortex was performed using SPECIAL (Mlynárik et al., 2006) with echo time of 2.8 ms, repetition time of 4 s. The volumes of interest (VOI) for MRS were 1.8 × 3.0 × 3.0 and 1.8 × 6.5 × 5.0 mm^3^ for the right hippocampus and cortex, respectively (Figure 1A). Typically, spectra were acquired in 40 blocks of 8 scans, which were summed after frequency alignment using the creatine peak.

Metabolite concentrations were determined with LCModel (Stephen Provencher Inc., Oakville, Ontario, Canada), including a macromolecule (Mac) spectrum in the database and using the unsuppressed water signal measured from the same VOI was used as internal reference. The following metabolites were included in the analysis: alanine (Ala), ascorbate (Asc), aspartate (Asp), creatine (Cr), γ-aminobutyrate (GABA), glutamine (Gln), glutamate (Glu), glutathione (GSH), glycine (Gly), glycerophosphorylcholine (GPC), glucose (Glc), lactate (Lac), *myo*-inositol (Ins), NAA, NAAG, phosphorylethanolamine (PE), phosphorylcholine (PCho), phosphocreatine (PCr), *scyllo*-inositol (scyllo), taurine (Tau). Given the negative correlation between phosphorylcholine and glycerophosphorylcholine in the LCModel fitting, their sum was quantified as total choline-containing compounds (GPC + PCho or tCho). Due to CRLB > 20% for most measurements, *scyllo*-inositol was considered to be below the detection limit and was excluded from the analysis (Figure 1).

Volume of the brain excluding olfactory bulb and cerebellum, and hippocampal volume were measured by manual segmentation of the T_2_-weighted images using ImageJ 1.37v (National Institutes of Health, United States).

### 2.3 Statistical analysis

Data are reported as mean ± SD. Correlations between memory performance and hippocampal volume or metabolite concentrations were analyzed using the Pearson correlation test.

## 3 Results

In this study, spectra had SNR of 37 ± 5 and 45 ± 6 in the hippocampus and cortex, respectively. Spectral linewidth estimated by LCmodel was 9.2 ± 1.8 Hz in the hippocampus and 13.0 ± 2.4 Hz in the cortex. Estimated metabolite concentrations (Figure 1) were in line with those previously determined by MRS in the rat cortex (Xin et al., 2010; Wang et al., 2012; Sonnay et al., 2016) and hippocampus (Duarte et al., 2009, 2019; Lanzillotta et al., 2024).

**Figure 2:**
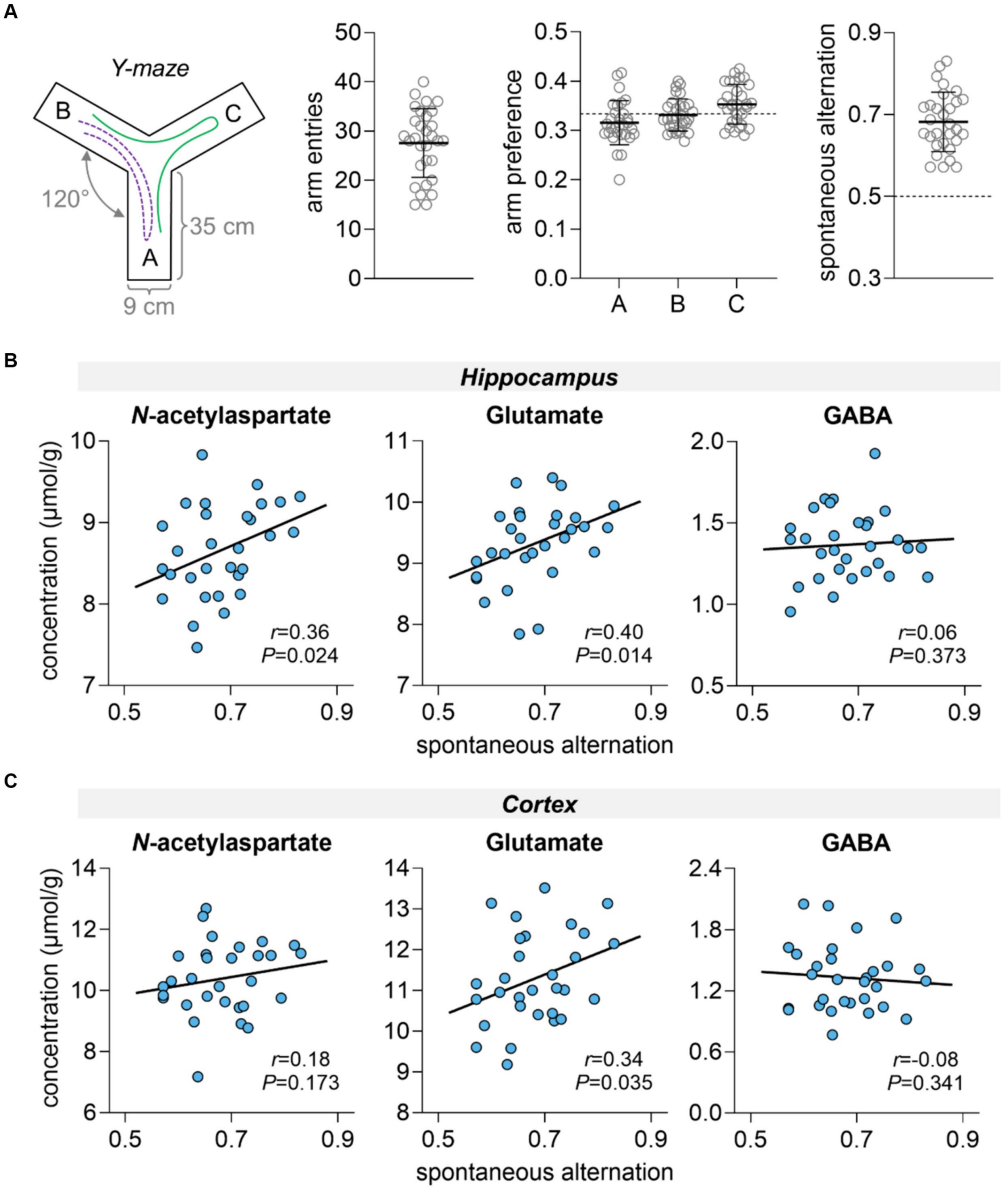
Spatial memory performance assessment in the Y-maze **(A)** and correlation with metabolite concentrations in the hippocampus **(B)** and cortex **(C)**. **(A)** Schematics of the Y-maze apparatus with three identical arms at a 120° angle from each other. During the test, the animal has free access to all three arms. If the animal chooses a different arm than the one it arrived from, this choice is called an alteration (example path represented by green line). While this is considered the correct response, returning to the previous arm is considered an error (example path represented by purple dashed line). Panel **(A)** shows the total number of entries in Y-maze arms, arm preference calculated as fraction of entries in each arm relative to the total number of arm entries, and fraction of spontaneous alternations relative to the total correct alternations possible. Dashed line at 50% represents the chance level, that is, memory impairment. Data is shown as mean ± SD of *n* = 30 overlaid on the symbols representing each individual rat.

Rats showed similar exploration of all Y-maze arms and mean spontaneous alternation in the Y-maze was 0.68 ± 0.07 (Figure 2A). Hippocampal-dependent spatial memory performance, as measured by the spontaneous alternation in the Y-maze, was positively correlated with hippocampal concentrations of NAA (*r* = 0.36, *p* = 0.024) and glutamate (*r* = 0.40, *p* = 0.014), but not with hippocampal concentrations of GABA (*r* = 0.06, *p* = 0.373) (Figure 2B). In the cortex, only concentrations of glutamate correlated with memory performance (*r* = 0.34, *p* = 0.035, Figure 2C).

Brain glutamate and NAA levels are often found to be correlated in rodents (Duarte et al., 2014) and humans (Kaiser et al., 2005), and thought to represent neuronal integrity (reviewed in Duarte et al., 2012). In the present study, correlation between glutamate and NAA was found in both cortex (*r* = 0.77, *p* < 0.001) and hippocampus (*r* = 0.63, *p* < 0.001).

Other trends for correlation between memory performance and neurochemicals in both cortex and hippocampus are shown in Table 1. Hippocampal lactate and cortical creatine correlated with memory performance at *p* < 0.05.

**Table 1:** Exploratory analysis of other potential correlations between metabolite concentrations and memory performance in the Y-maze.

	Hippocampus		Cortex	
	*r*	*p*	*r*	*p*
Glutamate	0.399	0.014	0.335	0.035
*N*-acetylaspartate	0.362	0.024	0.178	n.s.
*myo*-inositol	0.242	n.s.	−0.047	n.s.
Taurine	0.181	n.s.	−0.162	n.s.
Creatine	0.255	n.s.	0.376	0.020
Phosphocreatine	0.291	n.s.	−0.190	n.s.
Glucose	−0.207	n.s.	−0.012	n.s.
Glutamine	0.101	n.s.	0.071	n.s.
Aspartate	0.111	n.s.	−0.026	n.s.
Ascorbate	0.173	n.s.	−0.269	n.s.
Phosphorylethanolamine	−0.066	n.s.	0.059	n.s.
GABA	0.062	n.s.	−0.078	n.s.
Glutathione	0.153	n.s.	0.002	n.s.
*N*-acetylaspartatylglutamate	−0.229	n.s.	0.156	n.s.
Choline-containing compounds	0.207	n.s.	0.000	n.s.
Lactate	0.319	0.043	0.106	n.s.
Glycine	−0.183	n.s.	*	
Alanine	−0.060	n.s.	*	

n.s., non-significant at *p* < 0.05; * not analyzed due to substantial number of rats (>10%) with CRLBs > 20%.

**Figure 3:**
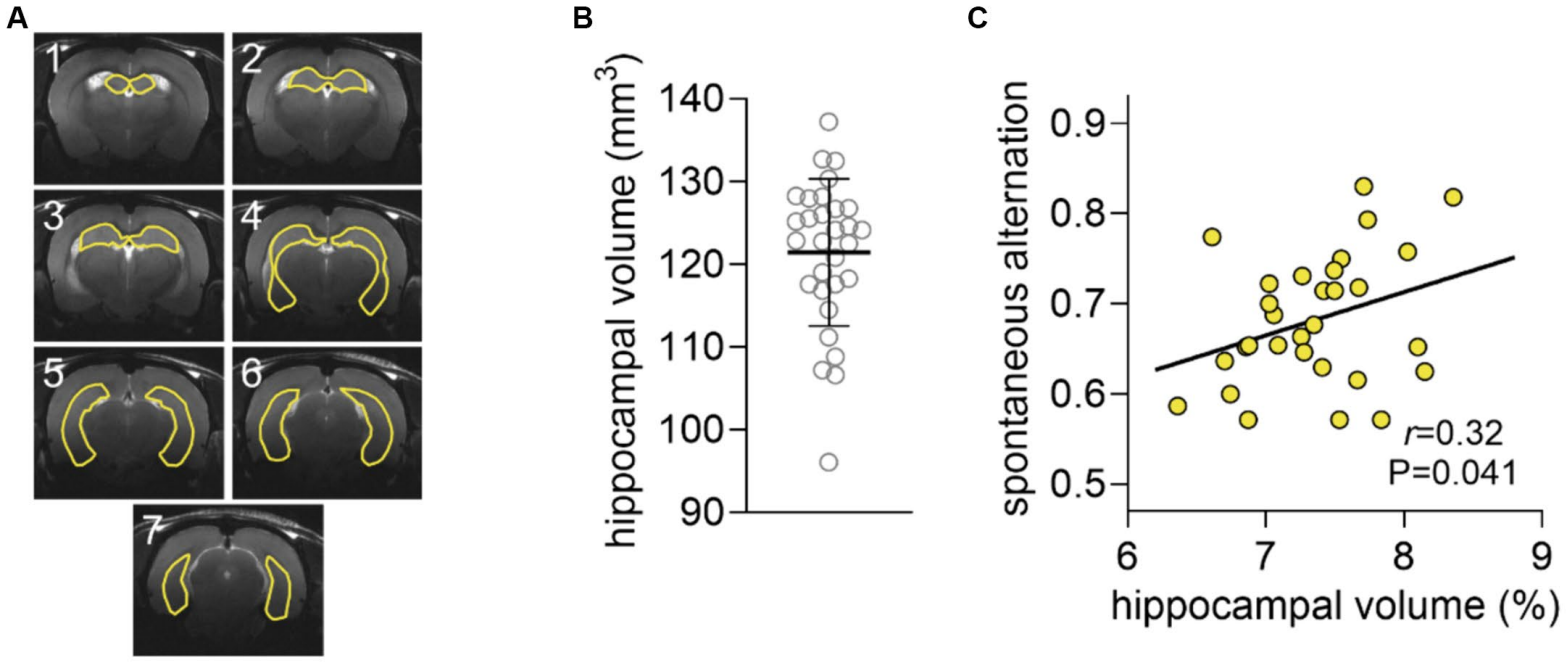
Analysis of hippocampal volume. **(A)** Representation of a typical segmentation of hippocampal volume overlaid on the respective anatomical MRI scan. The seven 0.8-mm consecutive that encompass the hippocampus are shown. **(B)** Distribution of the volume of the hippocampus shown as mean ± SD of *n* = 30 overlaid on the symbols representing each individual rat. **(C)** Spontaneous alternation in the Y-maze plotted against the volume of the hippocampus as fraction of that of the brain.

Hippocampal volume was 121.4 ± 8.9 μL and did not significantly correlate with spontaneous alternation (*r* = 0.23, *p* = 0.106, Figure 3). However, the correlation was significant when normalizing the volume of the hippocampus to that of the brain (*r* = 0.32, *p* = 0.041, Figure 3). Interestingly, the relative hippocampal volume correlated with the hippocampal concentration of NAA (*r =* 0.36, *p* = 0.026), but not glutamate (*r* = 0.29, *p* = 0.125) or GABA (*r =* 0.109, *p* = 0.294).

## 4 Discussion

While variations in brain metabolite levels have been associated to behavior impairment in pathology, the present study reveals that the variation in resting NAA and glutamate concentrations in the hippocampus are associated with performance in a hippocampal-dependent spatial memory task. Both NAA and glutamate showed a ~2 mmol/L concentration range in the rat hippocampus, and ~4 mmol/L in the cortex. This wide range of concentrations in the non-diseased brain is likely to be a functional corelate, as suggested by the present findings.

NAA is a widely accepted marker of neuronal density or integrity. NAA is synthetized in neuronal mitochondria and, therefore, energy metabolism impairments in neurons have been proposed to result in lowering of NAA levels (Duarte et al., 2012; Duarte and Xin, 2019). For example, treatment of neurons with mitochondrial toxins, such as 3-nitropropionic acid, results in depletion of neuronal NAA, which can recover after toxin withdrawal (Dautry et al., 2000). NAA accumulates in neurons and is mainly hydrolyzed in oligodendrocytes for metabolic support of myelin formation and, while the loss of neurons in neurodegenerative pathologies has been generally associated with reduced NAA levels, accumulation of NAA is observed upon oligodendrocyte dysfunction (Duarte et al., 2012). Given the synthesis and accumulation of NAA in neurons, the correlation between hippocampal NAA and a hippocampal-dependent task is not surprising.

Glutamate is most immediately formed in neurons from glutamine of astrocytic origin but can also be formed from the tricarboxylic acid cycle intermediate 2-oxoglutarate. Thus, glutamate in neurons constitutes a hub between energy metabolism and excitatory neurotransmission (Duarte and Xin, 2019; Andersen et al., 2021). The transamination reactions involving glutamate are key to the malate–aspartate shuttle that mediates the transfer of reducing equivalents from the cytosol into the mitochondrial matrix. Therefore, one can speculate that a larger concentration of glutamate provides a higher efficiency at relaying the metabolic activation of glycolysis during neuronal stimulation with the respiratory rate of mitochondria. Although neurons contain the largest brain pool of glutamate, astrocytes also contain significant amounts of this amino acid, and astrocytic pyruvate carboxylation is required for *de novo* synthesis of glutamate (Dienel, 2019; Rae et al., 2024). ^1^H MRS *in vivo* does not provide cellular resolution to determine whether astrocytic or neuronal glutamate correlates with memory performance. However, it is thought by many that activity-induced glycolytic activation mainly takes place in astrocytes (discussed in Dienel, 2019) and, in that case, one would expect that the astrocytic pool of glutamate is the one needed to maintain an efficient redox cycling (interconversion of the cytosolic cofactors NADH and NAD^+^) couped to activity-associated glucose utilization.

Given the close relation between these two metabolites and neuronal mitochondrial function, and the fact that spatial memory performance in the Y-maze task involves hippocampal metabolism (McNay et al., 2000, 2001), one might speculate that the association of NAA and glutamate with Y-maze performance reflects the capacity of neurons (or supporting astrocytes) to metabolically fuel synaptic function during exploration of the Y-maze. While it is surprising that levels of GABA were unrelated to Y-maze spontaneous alternation, GABAergic neurotransmission is less demanding than glutamatergic transmission in terms of energetic requirements (Duarte and Gruetter, 2013), which agrees with the notion of memory performance in this Y-maze task relating to mitochondrial metabolism in neurons.

Cortical glutamate concentrations were also correlated with spontaneous alternation in the Y-maze, which can be attributed to the fact that, although dependent on the hippocampus, performance in this task also involves some degree of cortical processing. In fact, during the Y-maze test, several brain circuits operating across various brain areas are recruited while walking and exploring the maze arms. The choice of the Y-maze spontaneous alternation as behavior task in our study was based on the fact that the hippocampus, but not the cortex, display metabolic activation during exploration of the Y-maze (McNay et al., 2000, 2001). The relative hippocampal volume (normalized to total brain size) was correlated with performance in the hippocampal-dependent task, suggesting a structure–function relation underlying hippocampal functioning in the Y-maze task.

In addition to NAA, glutamate and GABA, the remaining concentrations of the neurochemical profile were used in an exploratory manner, which risks identifying unreal associations. Despite analyzing 30 rats, this sample size is rather limited for robust investigation of all metabolites in the neurochemical profile, especially those with small amplitude signal in the spectra. Nevertheless, it is interesting to note that lactate levels in the hippocampus and creatine levels in the cortex were correlated with memory performance, suggesting a link between energy metabolism and brain function.

Cortical glycine and alanine were not used in this exploratory analysis because nearly half of the rats showed CRLBs above 20%. Increased CRLBs can result from either poor spectra in terms of SNR and linewidth, or real lower concentrations relative to the SNR level. The sensitivity to reliably determine the concentrations of these two amino acids could have been achieved by larger number of scans, although that would also implicate longer MRS acquisition times, as well as longer time under isoflurane anesthesia. In fact, the use of anesthesia in this study can be considered a limitation, since it is expected to alter neuronal activity and metabolism. Both glutamate and GABA (as well as NAA, total creatine, and many other metabolites in the neurochemical profile) have been reported to increase in the mouse brain during isoflurane anesthesia, when compared with isoflurane withdrawal in the presence of pancuronium bromide for immobilization (Boretius et al., 2013). Thus, performing MRS in awake rats, which have been trained to the MRS acquisition, is the best option to address the anesthesia bias in such MRS studies (Takado et al., 2022).

MRS detects the total metabolite pool in the volume of interest. The present finding that resting levels of glutamate but not GABA were associated with Y-maze performance is supported by measurements of extracellular neurotransmitters. Namely, in microdialysis studies, cognitive training in a rat model resulted in increased extracellular levels of glutamate but not GABA in the dentate gyrus of the hippocampus (Sheng et al., 2005), and the rat hippocampus also showed increased efflux of glutamate but not GABA during an object recognition task (Stanley et al., 2012). Additional evidence supports the importance of glutamate release for hippocampal function. For example, inhibited glutamate release in the dorsal hippocampus of rats was found to correlate with the spatial memory deficits after treatment with benzodiazepines and ethanol (Shimizu et al., 1998), and the genetic deletion of the cystine/glutamate antiporter X_C_^−^ resulted in lower extracellular glutamate and, at the same time, memory impairment in the Y-maze (De Bundel et al., 2011).

In sum, the present results directly suggest a relation between brain function and the concentrations of NAA and glutamate, which are most abundant detected by non-invasive MRS. This is an exploratory study, and only male rats were used because of sex differences in metabolite concentrations (Duarte et al., 2014). Furthermore, we have employed a simple behavioral task but further studies with a battery of behavioral tasks are warranted in future work.

## Data Availability

The original contributions presented in the study are included in the article/supplementary material, further inquiries can be directed to the corresponding author.
